# Improving precision of vaccine efficacy evaluation using immune correlate data in time-to-event models

**DOI:** 10.1038/s41541-024-00937-6

**Published:** 2024-11-11

**Authors:** Julie Dudášová, Zdeněk Valenta, Jeffrey R. Sachs

**Affiliations:** 1 Quantitative Pharmacology and Pharmacometrics, MSD, Prague, Czechia; 2 First Faculty of Medicine, Charles University, Prague, Czechia; 3 Institute of Computer Science of the Czech Academy of Sciences, Prague, Czechia; 4 Quantitative Pharmacology and Pharmacometrics, Merck & Co., Inc., Rahway, NJ USA

**Keywords:** Drug development, Predictive markers, Vaccines, Infectious diseases

## Abstract

Understanding potential differences in vaccine-induced protection between demographic subgroups is key for vaccine development. Vaccine efficacy evaluation across these subgroups in phase 2b or 3 clinical trials presents challenges due to lack of precision: such trials are typically designed to demonstrate overall efficacy rather than to differentiate its value between subgroups. This study proposes a method for estimating vaccine efficacy using immunogenicity (instead of vaccination status) as a predictor in time-to-event models. The method is applied to two datasets from immunogenicity sub-studies of vaccine phase 3 clinical trials for zoster and dengue vaccines. Results show that using immunogenicity-based estimation of efficacy in subgroups using time-to-event models is more precise than the standard estimation. Incorporating immune correlate data in time-to-event models improves precision in estimating efficacy (i.e., yields narrower confidence intervals), which can assist vaccine developers and public health authorities in making informed decisions.

## Introduction

Vaccine phase 3 trials are randomized double-blinded controlled clinical studies which aim to demonstrate the safety and efficacy of a vaccine. *Vaccine efficacy* (VE) is defined by means of relative risk of developing a disease (or other endpoint of interest) in vaccine and control (often placebo) arms^1^. The risk of disease in each arm is typically represented by incidence rate (IR) which is the ratio of counts of disease cases to person-years at risk. (In this work we refer to the typical method for VE estimation as case-counting.) Compared to many other drugs and biologics, vaccine phase 3 trials are particularly large (with sample sizes of tens of thousands of subjects) and lengthy (with observation periods of months to years) to accrue the safety data and the number of disease cases necessary to obtain a sufficiently precise case-count estimate of VE.

Vaccine immunogenicity and efficacy in a group (demographically defined sub-population) can depend on their *baseline covariates*. These covariates are demographic characteristics (e.g., age or pre-vaccination exposure to a wild-type virus) or other information (e.g., time and site of enrollment) collected before the time of randomization (the point at which a clinical trial subject is randomly assigned to vaccine or control arm of the study). Primary analyses generally do not consider baseline covariates (measured and unmeasured covariates are assumed to be balanced, on average, between the vaccine and control arms and to represent the population for which the vaccine is intended). However, incorporating prognostic baseline factors in the statistical analysis of clinical trial data (often referred to as *covariate adjusted analysis*, or simply *adjusted analysis*) can result in a more efficient use of data to demonstrate and quantify the effects of vaccination. This efficiency results from reducing the variability of vaccination effect estimation, which leads to narrower confidence intervals (CI) and more powerful hypothesis testing^2–4^. The benefits and methodological considerations of adjusted statistical analysis of clinical trial data have been recently described in the FDA guidance document^5^ *Adjusting for Covariates in Randomized Clinical Trials for Drugs and Biological Products*.

In addition to estimation of *overall* VE (from data for the whole enrolled population, a.k.a., marginal vaccination effect), estimation of VE in *subgroups* defined by baseline covariates (a.k.a., conditional vaccination effect) can be of interest. That is because baseline covariates may not only affect the clinical endpoint (the incidence of the disease can vary with age, for example), but they can also impact the vaccination effect on the clinical endpoint (VE can vary with, e.g., age). This phenomenon is referred to as vaccination effect heterogeneity^6^. Understanding the impact of covariates on VE is often key to decisions by vaccine developers and public health authorities. However, assessment of VE in demographic subgroups is typically challenging when case-count methods are used. Most vaccine phase 3 clinical trials are powered for overall VE as the primary endpoint and attempts to estimate VE in subgroups often lead to CI being too wide to reliably infer VE trends across those subgroups.

In most efficacy trials, subjects’ immune response post-vaccination (immunogenicity) is measured in addition to assessing their primary clinical endpoint. An immunogenicity biomarker which reliably predicts protection is called a *correlate of protection* (CoP)^7^. Immunogenicity value is not a baseline covariate (it is measured after randomization and should, in principle, be affected by vaccination and time since vaccination) and therefore would not be typically included in the adjusted analysis. However, immunogenicity meeting CoP criteria implies a significant association with VE and can thus increase precision of VE estimation (hereinafter referred to as *immunogenicity-based* estimation). This has been confirmed (by clinical trial simulations) to be true for both overall VE^8^ and VE in covariate-defined subgroups^9^.

A method for VE estimation using phase 3 clinical trial data on immunogenicity and binary clinical endpoint has been first proposed by Dunning^10^ and extended by Coudeville et al.^11^. The method has been further studied by several other authors^9,12–14^ and applied to numerous vaccines^8,15–19^. Recently, Dudasova, Valenta & Sachs^9^ used logistic regression to quantify the relationship between immunogenicity, covariates, and probability of disease (or of another binary clinical endpoint) and integrated that relationship with observed immunogenicity data to obtain estimates of VE in subgroups of interest. The subgroups are pre-specified prior to the analysis as one would typically do for an exploratory analysis of clinical trial data: the subgroups are typically based on demographic factors anticipated to impact immunogenicity and/or protection. The *vaxpmx* R package implementing the logistic regression-based approach has been made publicly available^20^.

The work presented here is motivated by a need to extend the method (for immunogenicity-based VE estimation in demographic subgroups) to account for the *time-to-event* character of right-censored clinical endpoint data: to enable estimations by means of hazard rates. The use of time-to-event data can bring benefits over use of the binary endpoint data, specifically when the factors affecting the disease occurrence vary with time over the course of the study. For example, IRs and virus type circulation can be highly variable and/or exhibit strong seasonal trends. In such cases, accounting for the time of vaccination and time of disease occurrence is key to appropriate data interpretation. Models for time-to-event data can also be applied to study the time-dependence (durability) of VE. This work uses statistical modeling of right-censored time-to-event and immunogenicity data to elucidate VE in covariate-defined subgroups.

The proposed methodology is illustrated with two vaccine examples: high-potency live-attenuated herpes zoster (shingles) vaccine (Zoster Vaccine Live, Merck Sharp & Dohme LLC, a subsidiary of Merck & Co., Inc., Rahway, NJ, USA; hereinafter referred to as *zoster vaccine*) and live attenuated tetravalent dengue vaccine (Dengue Tetravalent Vaccine, Live, CYD-TDV, Sanofi Pasteur, Inc.; herein after referred to as *dengue vaccine*). Efficacies of both vaccines have been previously shown to be affected by some baseline covariate (zoster vaccine by age group^21^, CYD-TDV vaccine by pre-vaccination natural dengue infection^22,23^).

## Results

Both examples presented below aim to illustrate a quantitative approach which can increase understanding of VE in demographic subgroups, and to do so early in development, even before case-count data from larger-scale (“pivotal” phase 3) studies become available. The examples use data (immunogenicity and a time-to-event clinical endpoint) from phase 3 sub-studies which are similar (in terms of sample size) to data sets often available from earlier, “proof-of-concept” phase 2b development trials.

We first study effects of baseline covariates on immunogenicity, as heterogeneity in immunogenicity can signal heterogeneity in efficacy. Next, we perform *immune correlates analysis* to verify the assumption that immunogenicity is associated with VE. This analysis includes correlate of risk (CoR) and CoP assessments. Both assessments are performed with time-to-event regression models; a CoR is defined by significant association between immunogenicity and clinical endpoint, while a CoP is (generally) a CoR which has insignificant effect of vaccination status when controlling for immunogenicity^24^. Finally, we integrate the best-fitting immunogenicity-risk model, possibly adjusted for baseline covariates, with observed immunogenicity data to obtain estimates of VE in subgroups of interest. The key demographic subgroups used in the examples here (based on pre-specified demographic factors anticipated to have potential impact) are age groups for the zoster vaccine and serostatus-based groups for the dengue vaccine.

Because both examples are of licensed vaccines for which data from additional larger studies are available, we can compare findings of our analysis with case-counting results from independent larger datasets as a qualification step.

### Zoster vaccine

Zoster vaccine is one of two currently available vaccines for the prevention of herpes zoster (HZ, shingles) which is caused by the reactivation of latent varicella-zoster virus (VZV). We use data collected in an immunogenicity sub-study to the phase 3 Shingles Prevention Study (SPS)^21^ to evaluate effect of age on zoster vaccine VE. This sub-study enrolled 1395 subjects and is described in detail by Levin et al.^25^. The immunogenicity biomarker considered in this analysis is the fold rise in VZV antibody titers, measured at baseline and week 6 post-vaccination by glycoprotein ELISA (gpELISA), as it has been previously shown to be a reliable predictor (non-mechanistic, relative CoP) for zoster vaccine VE^26^.

#### Age affects immune response post vaccination

**Fig. 1:**
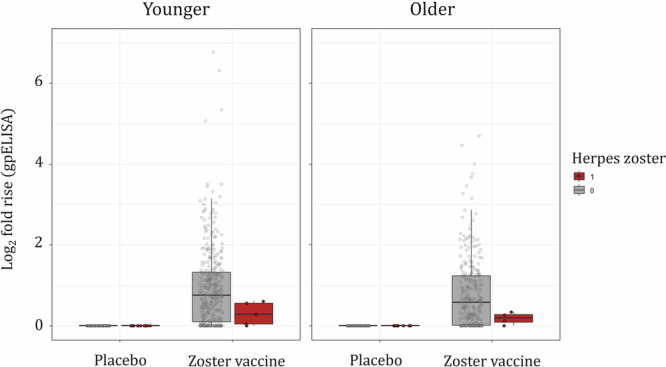
Zoster vaccine is generally less immunogenic in older subjects than in younger subjects. Distribution of log_2_ fold rise in VZV antibody titers (gpELISA) at baseline and week 6 (i.e., 6 weeks post vaccination) in HZ cases (red circles) occurring between week 6 and end of follow-up (~3 years), and non-cases including all participants who did not have HZ during the follow-up^25^. Boxplots show first quartile (lower edge of the box), median (horizontal line in the box), and third quartile (upper edge of the box), with participants stratified according to treatment assignment and HZ disease status; the whiskers indicate 1.5 times the interquartile range from the box. Red and gray circles show individual values of log_2_ fold rise in VZV antibody titers of HZ cases and non-cases, respectively.

Figure 1 summarizes the immunogenicity of younger ($\le$69 years) and older ($\ge$70 years) subjects. Values of log_2_ fold rise in VZV antibody titers measured at baseline and 6 weeks post vaccination by gpELISA were significantly higher in younger vaccinated participants than in older vaccinated ones (*p* value, 0.046), indicating lower vaccine-induced immune response in older subjects.

#### Fold rise in VZV antibody titers is a CoR

**Fig. 2:**
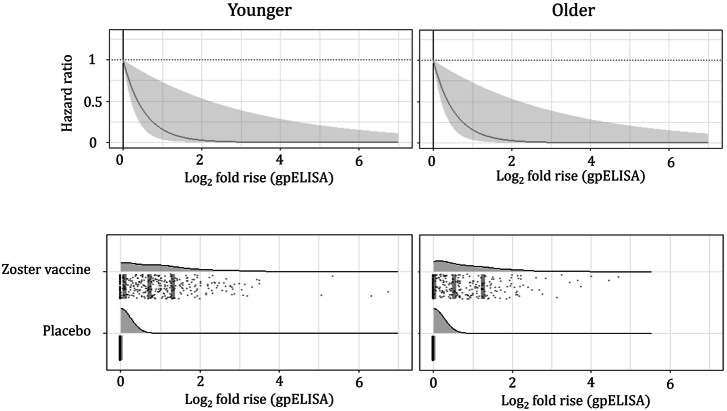
Estimated relationships between log_2_ fold rise in VZV antibody titers and hazard ratio (with median immunogenicity observation as the reference) of HZ, *ϱ(T).* Fold rise is an inverse correlate of risk (i.e., is significantly associated with hazard of HZ) in both younger and older subjects. Log_2_ fold rise is the only predictor in the selected model (Model a, Supplementary Table 1), risk curves for younger and older groups are therefore identical. The hazard ratio (y-axis) represents the hazard relative to the median of all immunogenicity data (shown with black vertical line) for which it equals to 1. The bottom plots show individual values of log_2_ fold rise by age group and vaccination status (gray points) with respective empirical probability density functions; gray vertical lines represent median, first quartile, and third quartile. For all placebo recipients, log_2_ fold rise is assigned to be 0 (see Methods); the density function shown in the plot for placebo recipients is an artifact of the smoothing function used by the smoothing kernel density estimator.

In the CoR analyses (Supplementary Table 1) we used a Cox proportional hazard (PH) model with immunogenicity represented either by a linear function or a quadratic polynomial of log_2_ fold rise. Even though the quadratic model (Model b, Supplementary Table 1) fit the data better than the linear model (AIC, quadratic, 426.0; AIC, linear, 428.0), the uncertainty in its coefficients was high (*p* values, 0.226 and 0.143), likely due to relatively small dataset (32 HZ cases), and the reduction in AIC is not enough to typically select a model with an additional parameter (e.g., via the Bayesian information criterion, BIC, often used for larger samples^27^). Thus, the simpler, linear model was selected for further analyses; log_2_ fold rise in this model was a statistically significant inverse correlate of risk (*p* value, 0.018), with estimated hazard ratios (HR) of 0.159 (95% CI, 0.035 to 0.729) in both younger and older subjects (Model a, Supplementary Table 1). Figure 2 shows estimated *risk curves (*$\varrho (T))$, i.e., HR per unit increase in immunogenicity ($T$), identical for younger and older participants, obtained from Model a (unadjusted for age). The PH assumption was satisfied for the selected model (*p* value of the association between scaled Schoenfeld residuals^28^ with time, 0.057, i.e., insignificant at statistical significance level $\alpha =\,$0.05). However, the relatively small (marginally significant) *p* value indicates potentially non-PHs, thus models allowing for time-varying coefficients may be considered in future work.

#### Fold rise in VZV antibody titers is a CoP (when assessed by the Prentice criterion)

Effect of vaccination status was insignificant when controlling for log_2_ fold rise in VZV antibody titers (Model j, Supplementary Table 1), satisfying the conditional independence criterion proposed by Prentice^24^ (see Methods for details) and indicating that the fold rise fully mediates the zoster vaccine-induced protection. Results of our analysis of zoster vaccine data complement and agree with previous findings^8,26^ that fold rise in VZV antibody titers (gpELISA) is a CoP against HZ.

#### Vaccine efficacy in younger and older subgroups is estimated more precisely with fold rise in VZV antibody titers

Immunogenicity-based zoster vaccine VE estimates (in younger and older groups; for HZ endpoint; using fold rise in VZV antibody titers and Cox PH Model a, Supplementary Table 1) are substantially more precise than case-count-based estimates (Table 1). The precision of VE is defined here as the width of the CI for a fixed population size and a fixed number of cases. The Cox PH model using data from only 1326 participants provided immunogenicity-based VE estimates with the lower limit of CI above 0 in both the younger (with 17 cases of HZ) and older (15 cases) subgroups and found a trend of higher VE in younger subjects (point estimates, 58% for younger, 53% for older). This can be contrasted with results based on case-counts alone, which did not indicate significant VEs in these subgroups and estimated that VE in younger subjects was lower than in the older group (point estimates of 55% for younger, 67% for older). The difference in VE predicted by the model (58% vs. 53%) was driven by the difference in immunogenicity between age groups and is not statistically significant.

**Table 1:** Case-count-based and immunogenicity-based estimates of zoster vaccine efficacy in younger and older subgroups of a cohort enrolled in the immunogenicity sub-study of SPS^25^

Age group	Control		Vaccinated		Vaccine efficacy, % (95% CI)			Results of immune correlates assessment	
	Person-years at risk	Cases	Person-years at risk	Cases	Case-count	Cox PH model using vaccination status^a^	Cox PH model using immunogenicity	CoR	CoP
Younger (≤69 years)	1059	12	983	5	**55 (–27 to 84)**	55 (–28 to 84)	**58 (21 to 68)**	✓	✓
Older (≥70 years)	654	11	728	4	**67 (–2 to 90)**	69 (1 to 90)	**53 (18 to 63)**		

^a^Typical model-based approach to estimating VE as 1-HR (comparing rates of herpes zoster among individuals who were immunized with zoster vaccine compared to those who were vaccinated with placebo) from model involving vaccination status, age group, and the interaction between vaccination status and age group as predictors of time-to-disease or end of follow-up. Widths of CI of the model-based estimates obtained by the typical approach (i.e., without the use of immunogenicity) are similar to those of case-count-based estimates.

Immunogenicity-based estimation for a given population size and number of cases is more precise (provides narrower confidence intervals) than case-counting when immunogenicity meets criteria for being a CoR and a CoP. Immunogenicity-based VE is reported for Cox PH model with log_2_ fold rise in VZV antibody titers (gpELISA) measured at baseline and week 6 (Model a, Supplementary Table 1).

#### Data from a larger study confirm the identified trend of lower VE in older participants

To qualify the estimated immunogenicity-risk model (Fig. 2), we compare the model-based VE with VE reported in a larger study (phase 3 SPS^21^ with 38546 subjects, 456 HZ cases in the younger group, and 501 HZ cases in the older group). The phase 3 VE is significantly different from 0 in both age groups and the trend of lower VE in older subjects (point estimates, 51% overall, 64% for younger, 38% for older) is consistent with model predictions, further supporting the claim of fold rise in VZV antibody titers being a CoP, i.e., predictive of VE, according to the definition by Plotkin & Gilbert^7^.

### Dengue vaccine

Dengue vaccine CYD-TDV was the first vaccine approved for prevention of dengue disease which is caused by infection with the dengue virus (DENV). There are four related but antigenically distinct serotypes (DENV1-4). We analyze recently published data from a cohort of 611 participants from the ancillary study^29^ to the phase 3 CYD-TDV trial in Asia (CYD-14)^30^. A model incorporating immunogenicity is used to estimate VE of CYD-TDV against virologically confirmed dengue (VCD) in seropositive and seronegative subgroups. We apply time-to-event models, under the competing risks paradigm (when observations are censored after the first VCD occurrence). For the DENV-Any VCD endpoint a Cox PH model is used. To model serotype-specific DENV1-4 VCD, cause-specific Cox PH^31,32^, and Fine-Gray subdistribution hazards^33^ models are applied. As an immunogenicity biomarker we use serotype-specific serum neutralizing (SN) antibodies, measured by plaque reduction neutralization test (PRNT_50_) 28 days post dose 3, as other studies found them to be predictive (relative CoP) of serotype-specific VCD^8,34–36^.

#### Serostatus affects immune response post vaccination

PRNT_50_ titers were significantly higher in seropositive vaccinated than in seronegative vaccinated for all serotypes (*p* values $<$ 1e-6), indicating that the CYD-TDV vaccine induced lower immune responses in seronegative subjects.

#### Average PRNT_50_ titer is a CoR for the DENV-Any, DENV2, DENV3, and DENV4 VCD endpoints

**Fig. 3:**
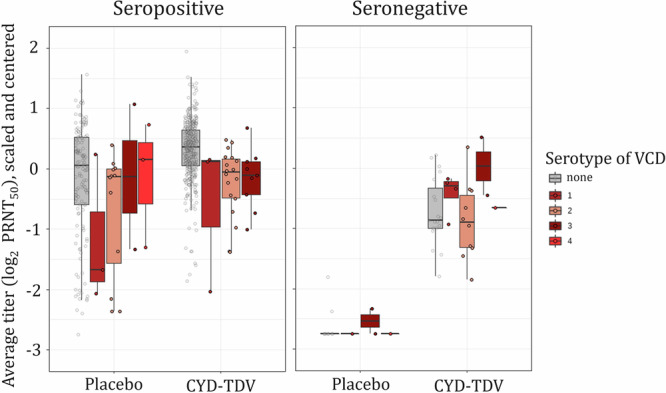
Immunogenicity of CYD-TDV, scaled and centered average log_2_ PRNT_50_ titers. Average titer was determined for each subject as the arithmetic average of respective four centered and scaled serotype-specific log_2_ PRNT_50_ titers. For each serotype, first, centering is performed by subtracting the mean log_2_ titer (across all subjects) from each log_2_ titer observation (per subject). Next, scaling is performed by dividing each (centered) log_2_ titer observation (per subject) by the standard deviation of log_2_ titers (across all subjects). Boxplots show first quartile (lower edge of the box), median (horizontal line in the box), and third quartile (upper edge of the box), with participants stratified according to treatment assignment and serotype-specific VCD endpoint; the whiskers indicate 1.5 times the interquartile range from the box. Red and gray circles show individual average antibody titers of serotype-specific VCD cases and non-cases, respectively.

**Fig. 4:**
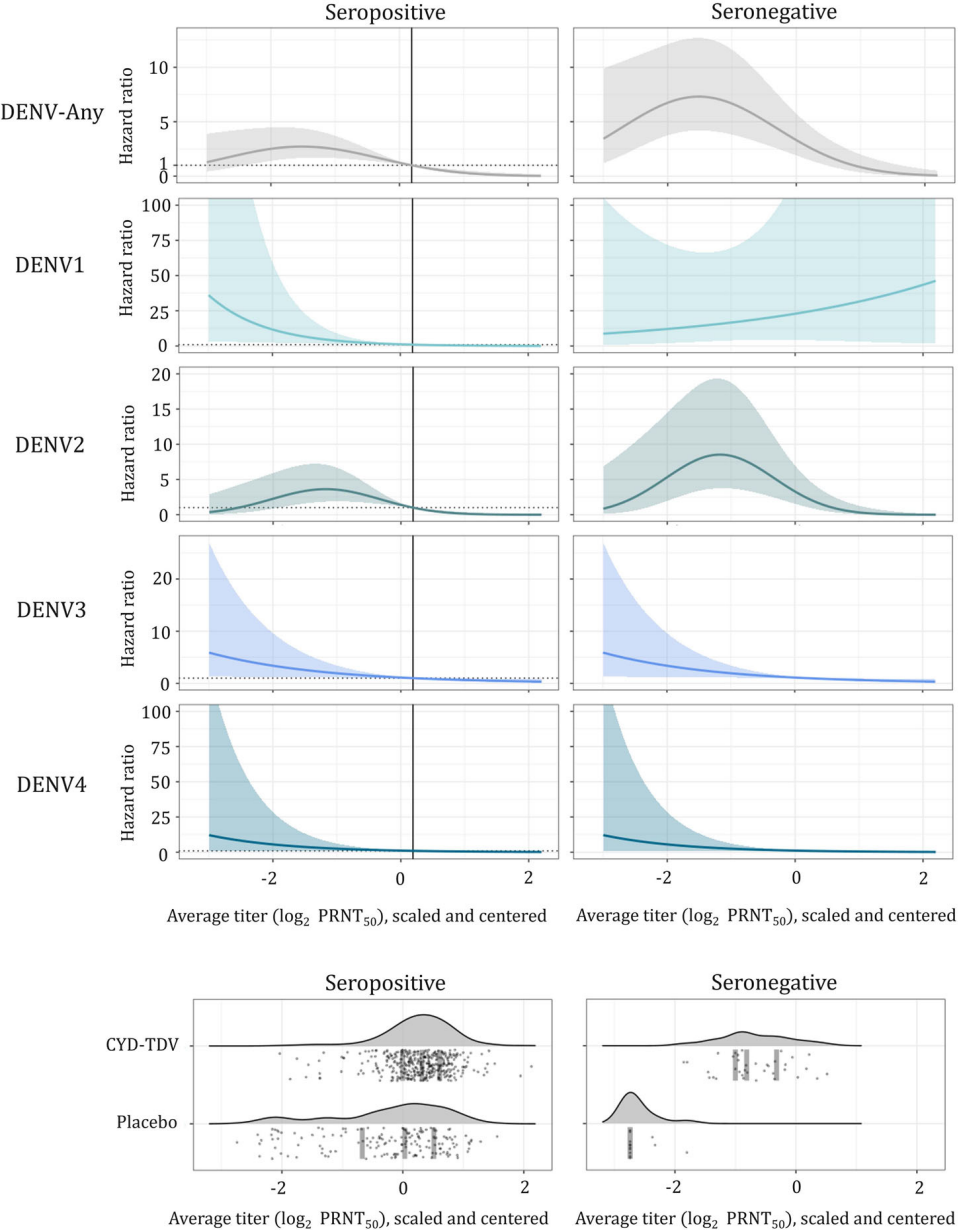
Estimated relationships between hazard ratio, *ϱ(T)*, and average log_2_ PRNT_50_ titer (with median immunogenicity observation as the reference) of serotype-specific VCD. Risk curves differ significantly between seropositive and seronegative subgroups for DENV-Any, DENV1, and DENV2. For DENV-Any and DENV2 the Cox PH model with quadratic term for average titer (non-monotonic risk curves) provided the best fit to data; the increase in risk with average titer is not significant, because confidence intervals include a constant curve over the titer range for non-monotonicity. The hazard ratio (y-axis) represents the hazard relative to that for a population with titer at the median of all immunogenicity data (observed in a seropositive subject and shown with black vertical line) for which (by definition) this ratio equals 1. The bottom plots show (gray points) individual participants’ values of average titer (averaged across serotypes after mean-centering and scaling by standard deviation) by serostatus (column) and vaccination status (row) with respective empirical probability density functions; gray vertical lines represent median, first quartile, and third quartile. For seronegative placebo recipients, the density function shown in the plot is an artifact of the smoothing function used by the smoothing kernel density estimator. (Models 0b-I, 1b-I, 2b-I, 3b-I, and 4b-I for DENV-Any and DENV1, 2, 3, and 4 endpoints, respectively, are visualized, see Supplementary Table 2 for details. The proportional hazards assumption was satisfied for all visualized models.).

Serotype-specific log_2_ PRNT_50_ titers for the four types were correlated. Variance inflation factors (VIFs)^37^ were 2.54, 2.42, 2.72, and 2.19 for serotypes 1–4, respectively, and pairwise correlation coefficients are shown in Supplementary Fig. 1. Thus, in the CoR analyses either average log_2_ PRNT_50_ titer (a single average value per subject of their four centered and scaled serotype-specific log_2_ titer values, Fig. 3) or the first two principal components (with cumulative proportion of variance of 84.4%) were used. Supplementary Table 2 shows general consistency in predictors identified as significant by models using average titer as the immunogenicity representation and models using immunogenicity principal component(s). Cause-specific Cox PH models provided consistent results with Fine-Gray subdistribution hazards estimators of serotype-specific VCD (Supplementary Table 2). Immunogenicity was a statistically significant correlate of risk in both seropositive and seronegative subjects for the DENV-Any, DENV2-4 VCD endpoints; and in seropositive subjects for DENV1 VCD endpoint. Figure 4 shows estimated risk curves *(*$\varrho (T))$, i.e., HR as a function of average log_2_ PRNT_50_ titer ($T$), for baseline seropositive and seronegative participants, obtained from Cox PH models.

#### Average PRNT_50_ titer is a reliable CoP (when assessed by the Prentice criterion) for the DENV-Any and DENV2 VCD endpoints

For overall and cause-specific Cox PH models (and when immunogenicity is represented by average titer) the effect (on $\varrho (T))$ of vaccination status was insignificant for the DENV-Any and DENV1-4 VCD endpoints (Table 2, Supplementary Table 3). The lack of residual effect of vaccination when controlling for immunogenicity (i.e., the Prentice criterion for a CoP) does not prove that the risk curves are the same for vaccinated and placebo subjects; the lack of a significant difference can be the result of either the absence of an effect or of the paucity of data (for example, DENV4 has only 1 VCD case in vaccine arm thus there is insufficient information about the risk curve in this arm). However, results of models with enough cases (DENV-Any, DENV2) indicate that DENV serum neutralizing antibody titers (average log_2_ PRNT_50_) are predictive of protection. The estimated risk curves are serotype-specific, and for DENV-Any and DENV1-2 they differ between seropositive and seronegative groups (as seen in the left versus right column in Fig. 4). Our findings support previous studies that pre-vaccination dengue serostatus influences the protectiveness of the neutralizing antibody response induced by vaccination^38^.

**Table 2:** Case-count-based and immunogenicity-based estimates of CYD-TDV dengue vaccine serotype-specific efficacy in seropositive and seronegative subgroups of a cohort enrolled in the Cebu ancillary study^29^ to the phase 3 CYD-TDV trial in Asia

Serostatus	Control		Vaccinated		Vaccine efficacy, % (95% CI)			Results of immune correlates assessment	
	Person-years at risk	Cases	Person-years at risk	Cases	Case-count	Cox PH model using vaccination status^a^	Cox PH model using immunogenicity	CoR	CoP
DENV-Any									
Seropositive	764	24	1808	35	**38 (−3 to 63)**	37 (−5 to 63)	**29 (14 to 43)**	✓	✓
Seronegative	53	5	121	17	**−48 (−280 to 42)**	−29 (−252 to 53)	**−14 (−136 to 47)**		
DENV1+									
Seropositive	764	3	1808	3	**58 (−109 to 91)**	56 (−116 to 91)	**63 (20 to 86)**	✓	✓
Seronegative	53	1	121	4	**−74 (−1420 to 80)**	−37 (−1126 to 85)	**−87 (−1404 to 70)**		
DENV2									
Seropositive	764	12	1808	18	**37 (−31 to 69)**	36 (−33 to 69)	**32 (12 to 51)**	✓	✓
Seronegative	53	0	121	10	**−Inf (−Inf, NA)**	−Inf (−Inf to 100)	**−187 (−1096 to 28)**		
DENV3									
Seropositive	764	2	1808	9	**−90 (−778 to 59)**	−95 (−805 to 58)	**32 (4 to 59)**	✓	✓
Seronegative	53	2	121	2	**57 (−201 to 94)**	73 (−198 to 98)	**64 (18 to 84)**		
DENV4++									
Seropositive	764	3	1808	0	**100 (NA, 100)**	100 (−Inf to 100)	**45 (2 to 78)**	✓	✓
Seronegative	53	1	121	1	**57 (−582 to 97)**	60 (−536 to 98)	**76 (10 to 94)**		

^a^Typical model-based approach to estimating VE as 1-HR (comparing rates of VCD among individuals who were immunized with dengue vaccine compared to those who were vaccinated with placebo) from model involving vaccination status, serostatus, and the interaction between vaccination status and serostatus as predictors of time-to-disease or end of follow-up. Widths of CI of the model-based estimates obtained by the typical approach (i.e., without the use of immunogenicity) are similar to those of case-count-based estimates.

+ Average titer was significantly associated with risk of DENV1 VCD in seropositive subjects only (when evaluated using cause-specific Cox PH model, which indicated a significant modification effect of serostatus on the effect of immunogenicity). Adding vaccination status as a predictor did not result in a significant improvement in fit, i.e., satisfied the Prentice CoP criterion.

++ The cause-specific hazard ratios corresponding to average titer for DENV4 VCD (which produced the VE estimates reported in this table, see Fig. 4) were independent of vaccination status. However, with so few cases of DENV4 VCD, the reason for meeting Prentice criterion might only be lack of data, rather than lack of vaccination effect.

Immunogenicity-based estimation for a given population size and case-count is more precise (provides narrower confidence intervals) than case-counting when immunogenicity meets criteria for being a CoR and a CoP. When immunogenicity in a subgroup is not significantly associated with risk of disease, the confidence interval of immunogenicity-based estimation can be as wide as that of case-counting for that subgroup (the case of DENV1 seronegative group). Immunogenicity-based VE is reported for Cox PH models with average titer (Models 0b-I, 1b-I, 2b-I, 3b-I, and 4b-I for DENV-Any and DENV1, 2, 3, and 4 endpoints, respectively, see Supplementary Tables 2 and 4 for details).

#### Vaccine efficacy in seropositive subgroup is estimated more precisely using average PRNT_50_ titer, consistently across serotypes

Immunogenicity-based CYD-TDV dengue vaccine VE estimates are reported and compared to case-count-based estimates in Table 2. For completeness (and as a sensitivity analysis), the additional models used for immunogenicity-based VE estimation (using principal component(s), and/or Fine-Gray method) are listed in Supplementary Table 4. VE estimates are generally consistent across all methods used. The Cox PH models with average titer as a predictor of VCD risk using data from 564 participants provided VE significantly different from 0 in the seropositive subjects against DENV-Any (with 81 cases of VCD), DENV1 (11 cases), DENV2 (40 cases), DENV3 (15 cases), and DENV4 (5 cases). In seronegative subjects, model-predicted VEs were negative for DENV-Any, DENV1-2, and positive with lower bounds of VE CI below 25% for DENV3-4 endpoints. The model predictions in seropositive subjects are substantially more precise than case-count-based estimation, which did not indicate significant VE against any endpoint.

#### Data from larger studies confirm the identified trend of lower VE in seronegative participants

In their pooled analysis of three larger studies, Sridhar et al.^22^ reported a VE (for DENV-Any) in seropositive subjects of 73% (95% CI, 59% to 82%), and a VE not significantly different from 0 in seronegative subjects, 32% (95% CI, −9% to 58%). This analysis leveraged data from three large trials enrolling 35146 subjects in total; with 758 cases in the seropositive group, and 500 cases in the seronegative group. (The three trials were the phase 3 CYD-14 in the Asia-Pacific region^30^, phase 3 CYD-15 in Latin America^39^, and phase 2b CYD-23 in Thailand^40^.)

Consistency between the case-count-based estimates for DENV-Any VCD endpoint (using the pooled dataset) and our immunogenicity-based predictions (using only a small subset thereof) shows: (i) the PRNT_50_ assay is predictive of CYD-TDV vaccine-induced protection (i.e., additional evidence that SN antibody titer is a CoP, beyond the finding that DENV-Any risk curve is independent of vaccination status), and (ii) the value of the proposed method in vaccine development (i.e., the ability to produce accurate (unbiased) and precise estimates of VE immunogenicity, when the assay is sufficiently predictive of protection, in a relatively small study).

## Discussion

We proposed an extension of the previously published^8,9^ *immunogenicity-based vaccine efficacy estimation*. The extension accounts for *time-to-event endpoints* in regression models. Such models for time-to-event data can be leveraged to (i) test the Prentice CoP criterion of the conditional independence of the risk of disease on the vaccination status, given the immunological endpoint; (ii) predict VE with more precision using the immunological endpoint; and (iii) assess, with more confidence, which, if any, baseline covariates impact VE. The proposed approach to the immune correlates analysis and the assessment of impact of demographic factors on VE was illustrated with zoster vaccine and dengue vaccine data from relatively small sub-studies to phase 3 trials.

We demonstrated with data from two vaccine clinical trials that immunogenicity-based VE estimation can substantially improve understanding of potential differences in VE between subgroups. This is possible when an immunogenicity biomarker is a CoP and is used in a time-to-event model, as it yields a narrower CI on the VE estimate (compared to case-counting on the same data).

Here, due to small sample sizes, neither case-count-based nor immunogenicity-based estimates demonstrated statistically significant VE differences between subgroups. However, the immunogenicity-based VE estimates were substantially more precise (had narrower CIs) when compared to case-counting, revealing not only *potential clinically significant differences* between the groups (Table 2), but also revealing potentially impactful (i.e., high enough) VE that was otherwise obscured by variability (Table 1).

The aim of this study was not to provide definitive answers on heterogeneity in efficacy for these two vaccines, but rather to describe and illustrate a quantitative approach enabling useful insights and early signals of this phenomenon leveraging only data that are routinely collected before or during phase 3 clinical trials. For a given sample size (i.e., number of participants and of cases), this immunogenicity-based approach provides a higher probability of identifying statistically significant differences than case-counting.

The immunogenicity biomarkers used in our analysis (for both vaccines) were shown to be predictive of VE as demonstrated in the qualification step of our analysis (i.e., comparing the predictions with case-count-based observations in independent and substantially larger studies). These biomarkers (fold rise in gpELISA antibodies for the zoster vaccine and PRNT_50_ titers for the dengue vaccine) were also shown to be CoP (for most endpoints) by the Prentice test.

Several limitations of the Prentice framework have been described in the literature, including a limited ability to test the criterion of conditional independence in the context of high VE, when few or no case data are available in the vaccine arm^13,41^. Thus, some authors rely on other approaches^41–48^ to evaluate the extent to which an immune biomarker allows for a correct VE prediction. In this work, the adoption of the Prentice framework is motivated by its widespread use and simplicity. Furthermore, to supplement its findings and address certain limitations, immunogenicity-based VE predictions are provided.

Our approach combines the advantages of traditional CoP assessment using the Prentice criterion (assessment of the ability to quantify risk based on immunogenicity without considering vaccination status, and the degree to which that can be done) and causal mediation analysis (assessment of causal effects and enabling predictions even if immunogenicity does not fully mediate the vaccine effect).

When VE is predicted using a regression model as outlined in this work, the degree of consistency between that prediction and the case-count-based VE estimate can be directly assessed. To account for the variability and uncertainty, any such comparisons should be based on not only point estimates but also on their CI. Thus, the larger the phase 3 trial that is leveraged for case-count VE evaluation, the more reliable the resulting conclusions about a CoP (assuming the populations are sufficiently similar). The consistency between model-based VE derived from one dataset and case-count-based VE observed in an independent dataset (or the same dataset if it is sufficiently large to provide a precise case-count VE) can serve as important supportive evidence of the immune biomarker being a CoP (i.e., predictive of VE). However, results of immune correlates analysis need to be interpreted cautiously while accounting for specifics of the studied disease, vaccine, and dataset: evaluation of immune correlates often require synthesis of multiple levels of evidence (obtained from, e.g., analyses of independent datasets from non-clinical experiments, epidemiological observations from natural infection studies, different trials for the same vaccine, and/or for different vaccines).

When a CoP (or, in some cases, a CoR) is established, the presented modeling approach provides a method of predicting efficacy of a new vaccine in different (real or hypothetical) populations before any cases are accrued in those populations. Such predictions can inform decisions in vaccine discovery and clinical development (e.g., go/no-go decisions, reformulation strategy, booster strategy) as well as post-approval decisions by public health authorities (e.g., decisions based on risk-benefit profile, vaccine recommendations, modifications to immunization schedules).^8,10,45,47–50^

In this work, we treat the immunogenicity data as a time-fixed predictor by using the immune biomarker value measured shortly post vaccination in the regression. For some vaccines, the biomarker value at the time of exposure (instead of at a fixed time) may be a more useful CoP^45,47,51^. Future work should evaluate the potential for predicting durability of efficacy using immunogenicity as a time-dependent predictor.

Time-to-event models used in this analysis (i.e., Cox PH and Fine-Gray models) assume proportionality of hazards. However, effects of immunogenicity, vaccination status, and baseline covariates on clinical outcome can theoretically vary with respect to time. The proportionality of hazards assumption should be therefore always verified and, if violated, other, more flexible time-to-event models^52–54^ should be used.

If an immune biomarker is a CoP (absolute or relative), the time-to-event regression models provide substantially more precise estimates of VE than case-counting when observed immunogenicity data in vaccine and control arms are used as input. This is key especially in (i) analyses of data from smaller trials (e.g., phase 2b proof of concept studies which are typically not powered for the efficacy endpoint CI lower bounds required of pivotal trials), and (ii) in subgroup analyses in phase 3 trials when understanding potential demographic differences in VE (e.g., age or prior exposure) can be key to public health decisions.

## Methods

### Data

#### Zoster vaccine

The immunogenicity sub-study of a double-blind, placebo-controlled efficacy trial of a high-potency live-attenuated herpes zoster (shingles) vaccine (zoster vaccine live) enrolled 1395 subjects. There were 32 cases of herpes zoster (HZ) between vaccination and end of follow-up. Blood samples obtained at baseline (prior to vaccination), 6 weeks after vaccination, and at 1, 2, 3 years thereafter were tested (among other assays) for VZV antibody titers by glycoprotein ELISA (gpELISA).

Information needed for this analysis (vaccination status, disease status, time to disease or end of follow-up, age, and VZV antibody titers prior to vaccination and at week 6) was available for 1326 subjects. These included 655 zoster vaccine recipients and 671 placebo recipients; 773 individuals were 60–69 years of age, 553 individuals were 70–93 years of age; and 32 individuals experienced HZ after the week 6 visit. Time-to-event was determined for each subject as the time after the week 6 visit to either HZ (for cases) or end of follow-up (for non-cases). Median time-to-HZ was 714 days (time range, 63–1191 days), median time-to-event (including both cases and non-cases) was 949 days (time range, 17–1259 days). Fold rise in VZV antibody titers is derived from the ratio of gpELISA measurements at week 6 divided by that prior to herpes zoster vaccination. Subjects with values of fold rise less than 1 are assigned fold rise value of 1 (log_2_ fold rise equal to 0; any reduction of assay value is likely the result of measurement error). All placebo recipients are also assigned fold rise value of 1 (log_2_ fold rise equal to 0; the control group has, by definition, no change of assay; any measured negative or positive change of assay value is likely the result of measurement variability).

Post-vaccination immunogenicity responses are visualized as boxplots by HZ status, vaccination status, and age group. The two-sided Mann-Whitney U test is performed to assess the difference between immunogenicity of herpes zoster vaccine in younger and older subjects.

#### Dengue vaccine

Anonymized data analyzed in this work were published and described in detail elsewhere^29^. Briefly, the ancillary study to the phase 3 CYD-TDV trial in Asia (CYD-14)^30^ was conducted in Cebu, Philippines; a cohort of participants ($n=$ 611, consisting of 417 CYD-TDV recipients and 194 placebo recipients; mean age, 8 years at baseline; age range, 2–14 years; 90% individuals were seropositive at baseline) were followed for more than 6 years after their third dose^29^. There were 87 VCD (DENV1, $n=\,$12; DENV2, $n=\,$43; DENV3, $n=\,$16; DENV4, $n=$ 6; unknown serotype, $n=$ 10) between the third dose of the vaccine and end of follow-up. Plaque reduction neutralization test (PRNT_50_) was conducted on blood samples collected shortly post vaccination (around day 28) and then annually and during symptomatic infections^29,55,56^.

In this work, for six individuals experiencing multiple VCDs (which are here considered competing risks) during the observation period (between the day 28 visit and the end of follow-up), only the first VCD is used. There were 81 VCD cases (DENV1, $n=\,$11; DENV2, $n=\,$40; DENV3, $n=\,$15; DENV4, $n=$ 5; unknown serotype, $n=$ 10) in the resulting dataset for models with DENV-Any endpoint. Time-to-event was determined for each subject as the time after the day 28 visit to either VCD (for cases) or end of follow-up (for non-cases). Median time-to-VCD was 385 days (time range, 1–2291 days), median time-to-event (including both cases and non-cases) was 1812 days (time range, 1–2291 days). Serostatus is a binary indicator of pre-vaccination exposure to at least one serotype of DENV. Immunogenicity biomarkers used in this analysis are serotype-specific log_2_ PRNT_50_ values measured around day 28 post the third dose of CYD-TDV (median time, 30 days; time range, 19–41 days). These log_2_ PRNT_50_ values were available for 564 subjects (392 CYD-TDV recipients and 172 placebo recipients). Average titer is a derived variable, a single summary biomarker that can be assessed as an immune correlate for DENV-Any and DENV1-4 endpoints. When serotype-specific PRNT_50_ titers are correlated (as here, see Supplementary Fig. 1), the average titer will typically have greater precision than analyses using models specific to each serotype. Average titer is determined for each subject as the arithmetic average of respective four centered and scaled serotype-specific log_2_ PRNT_50_ titers. For each serotype, first, centering is performed by subtracting the mean log_2_ titer (across all subjects) from each log_2_ titer observation (per subject). Next, scaling is performed by dividing each (centered) log_2_ titer observation (per subject) by the standard deviation of log_2_ titers (across all subjects).

Post-vaccination immunogenicity responses are visualized (Fig. 3, Supplementary Fig. 2) as boxplots by serotype-specific VCD status, vaccination status, pre-vaccination serostatus and serotype. The two-sample two-sided t-test is performed to assess the difference between immunogenicity of CYD-TDV in seropositive and seronegative subjects. The assumption of normality was satisfied for DENV1, DENV3, and DENV4, as confirmed visually using quantile-quantile plots and verified through the Kolmogorov-Smirnov test for both seropositive and seronegative groups. To assess the homogeneity of variances between groups, the two-sided Bartlett’s test was conducted. In the case of DENV4, where the assumption of homogeneity of variances was violated, the two-sided Welch’s t-test was employed as an alternative to the standard t-test. Additionally, for DENV2, where the assumption of normality was violated in the seropositive group, the two-sided Mann-Whitney U test was applied.

### Correlate of risk

An immune response biomarker is a *correlate of risk* (CoR) if probability of disease is statistically associated with biomarker levels. In the terminology defined by Qin et al.^57^, CoR uses data exclusively from subjects who have received active vaccination. However, in our analyses, we use data from all subjects (i.e., including those who received placebo control) in time-to-event models to evaluate immunogenicity as a CoR^8,9^. We thus aim to provide more comprehensive and precise assessment of the association between immunogenicity and the risk of disease.

Where there are multiple immune response biomarkers (serotype-specific log_2_ PRNT_50_ values for the dengue CYD-TDV vaccine), multicollinearity is assessed using VIFs (variance inflation factors) and pairwise Pearson’s correlation coefficients. Use of highly correlated immunogenicity predictors in regression is avoided by transforming them into new predictor(s): a single summary biomarker (e.g., average log_2_ PRNT_50_ titer for the dengue CYD-TDV vaccine), or principal component(s).

In *Cox PH models*, given a set of $L$ predictors ${x}_{i1},\,{x}_{i2},\,\ldots ,\,{x}_{{iL}}$ for subject $i$, the hazard function $\lambda (t{{|}}{X}_{i})$ has the form:

1 $$\lambda \left(t|{X}_{i}\right)={\lambda }_{0}\left(t\right)\mathrm{exp}\left({\beta }_{1}{x}_{i1}+{\beta }_{2}{x}_{i2}+\cdots +{\beta }_{L}{x}_{{iL}}\right)={\lambda }_{0}\left(t\right)\mathrm{exp}\left({lp}\right)$$

with baseline hazard ${\lambda }_{0}\left(t\right)$ identical for all subjects (has no dependency on $i$), and linear predictor ${lp}={\beta }_{1}{x}_{i1}+{\beta }_{2}{x}_{i2}+\cdots +{\beta }_{L}{x}_{{iL}}$. Parameters of the model ${\beta }_{1},\,{\beta }_{2},\,\ldots ,\,{\beta }_{L}$ can be estimated by maximizing the partial log-likelihood^58^.

Alternatively, a hazard function involving an interaction term (denoted, e.g., ${\beta }_{1,2}$) between, e.g., predictors ${x}_{1}$ and ${x}_{2}$ may be described as

2 $$\lambda \left(t|X\right)={\lambda }_{0}\left(t\right)\mathrm{exp}\left({lp}+{\beta }_{1,2}{x}_{1}{x}_{2}\right)$$

The effect of immunogenicity ($T$) can be estimated using either a linear term,

3 $$\lambda \left(t|T\right)={\lambda }_{0}\left(t\right)\mathrm{exp}\left({\beta }_{1}T\right)$$

or a non-linear term (or terms), e.g.,

4 $$\lambda \left(t|T\right)={\lambda }_{0}\left(t\right)\mathrm{exp}\left({\beta }_{1}\log (T)\right)$$

5 $$\lambda \left(t|T\right)={\lambda }_{0}\left(t\right)\mathrm{exp}\left({\beta }_{1}\sqrt{T}\right)$$

6 $$\lambda \left(t|T\right)={\lambda }_{0}\left(t\right)\mathrm{exp}\left({\beta }_{1}T+{\beta }_{2}{T}^{2}\right)$$

We define the *risk curve*, $\rho (T)$, as hazard ratio, a function of immunogenicity ($T$), representing the hazard relative to the reference datapoint (${T}_{\text{ref}}$).

7 $$\rho \left(T\right)=\,\frac{\lambda \left(t|T\right)}{\lambda \left(t|{T}_{\text{ref}}\right)}$$

In this work, models using the relevant immunogenicity biomarker are first fitted with linear and non-linear term(s) (Eqs. 3 and 6, resp.). The only predictor in these models is the immunogenicity biomarker, $T$. The best-fitting model is selected based on Akaike information criterion (AIC)^59^. Next, the resulting model is fitted adjusting for all pre-selected potentially clinically meaningful covariates^4,5,60–62^ and their interactions. The final model for CoR assessment is selected based on AIC (unadjusted or adjusted). The final model’s hazard ratio is visualized as one overall risk curve (for unadjusted model, e.g., for the zoster vaccine) or multiple risk curves (for adjusted models; one per each covariate realization value, e.g., seropositive and seronegative for the dengue CYD-TDV vaccine, or one per each combination of covariates’ realizations) with pointwise 95% CI. The studied immunogenicity biomarker is deemed a CoR if the coefficient involving the biomarker in the final model is different from 0 at pre-specified level of statistical significance (here we adopt $\alpha =0.05$).

In the context of competing risks^63^ (e.g., virologically confirmed dengue disease caused by DENV1-4), the effect of immunogenicity on time-to-event outcome is evaluated analogously using cause-specific Cox PH models^31,32^ and Fine-Gray subdistribution hazards models^33^, as further explained below.

*The cause-specific Cox PH model* has the form of:

8 $${\lambda }_{c}\left(t|{X}_{i}\right)={\lambda }_{0{\rm{c}}}\left(t\right)\mathrm{exp}\left({\beta }_{1{\rm{c}}}{x}_{i1}+{\beta }_{2c}{x}_{i2}+\cdots +{\beta }_{{Lc}}{x}_{{iL}}\right)$$

This PH model of event type $c$ at time $t$ allows effects of the covariates to differ by event types (e.g., DENV1-4), as the subscripted beta coefficients suggest. The cause-specific approach has a limitation in that it assumes *noninformative* censoring for subjects who experience competing events. This assumption is equivalent to saying competing events are independent, and the sum of individual event probabilities is generally not constrained to 1. However, there is no way to explicitly test whether this assumption is satisfied for any given dataset.

*The Fine-Gray subdistribution hazards model* aims at modeling the hazard function derived from a cumulative incidence function (CIF, also known as subdistribution hazard):

9 $${\lambda }_{c,\text{CIF}}\left(t|{X}_{i}\right)=-d\mathrm{ln}\{1-{\text{CIF}}_{c}\left(t|{X}_{i}\right)\}/{dt}$$

The CIF, accounting for competing risks, estimates the marginal probability for each competing event as a function of its cause-specific probability and overall survival probability (i.e., ensures the probabilities add up to 1). The CIF for event type $c$ at time ${t}_{J}$ is the cumulative sum up to that time of incidence probabilities over all times for the events of interest (i.e., type $c$), which is expressed as:

10 $${\text{CIF}}_{c}\left({t}_{J}{{|}}{X}_{i}\right)=\mathop{\sum }\limits_{j=1}^{J}\hat{{I}_{c}}\left({t}_{j}{{|}}{X}_{i}\right)=\mathop{\sum }\limits_{j=1}^{J}\hat{S}\left({t}_{j-1}{{|}}{X}_{i}\right)\times \hat{{\lambda }_{c}}({t}_{j}{{|}}{X}_{i})$$

where $\hat{S}\left({t}_{j-1}|{X}_{i}\right)$ denotes the estimate of overall (any event) probability of surviving ${t}_{j-1}$ (the last event-of-interest time before ${t}_{j}$) given predictor vector ${X}_{i}$ and $\hat{{\lambda }_{c}}({t}_{j}|{X}_{i})$ represents the estimate of hazard at ordered event time ${t}_{j}$ for the event-type of interest given ${X}_{i}$.

This approach overcomes the limitation of the cause-specific Cox PH approach by taking into account the *informative* nature of censoring due to competing risks (i.e., events other than the one of interest which alter the probability of experiencing the event of interest). The CIF models developed by Fine and Gray are analogous to the Cox PH model:

11 $${\lambda }_{c,\text{CIF}}\left(t|{X}_{i}\right)={\lambda }_{0c,{\rm{CIF}}}\left(t\right)\mathrm{exp}\left({\gamma }_{1}{x}_{i1}+{\gamma }_{2}{x}_{i2}+\cdots +{\gamma }_{L}{x}_{{iL}}\right)$$

The results from fitting these models have a similar interpretation regarding the effects of predictors in the model as can be derived from the Cox PH model approach for competing risks data.

### Correlate of protection

An immune response biomarker that is predictive of VE is termed a *correlate of protection* (CoP)^7^. Here, the Prentice criterion^24^ of conditional independence is applied to test if immunogenicity fully mediates the vaccine effect, i.e., if the effect of vaccination status on time-to-disease endpoint is insignificant after controlling for the impact of immunogenicity.

To assess a CoP, an additional predictor, the vaccination status, is added to the final CoR model (selected as described in the previous section). The studied immunogenicity biomarker is deemed a CoP if the coefficient involving the biomarker remains significantly different from 0 and the coefficient involving the vaccination status is *not* significantly different from 0 at pre-specified level of statistical significance (we adopt $\alpha =0.05$).

For an efficacious vaccine, a CoP should be also a CoR. However, as VE can differ across subgroups, a vaccine that is efficacious in some subgroups can also fail to be efficacious in others. In such a case, an immune biomarker could be a CoR in some subgroups only (and fail to be a CoR in others), but if it fully mediates the vaccine effect in the covariate-adjusted CoR model, we call it a CoP.

### Immunogenicity-based vaccine efficacy estimation

For illustration, assume that only one binary baseline covariate (e.g., age group or serostatus), $$X$$, is considered and VE is to be estimated in two covariate-defined subgroups (as in the presented examples). Let $${T}_{i}^{\text{vaccinated}}$$, $${T}_{j}^{\text{vaccinated}}$$ be the immunogenicity biomarker measurement for $$i$$-th vaccinated subject ($$i=1,\ldots ,\,I$$) in the first covariate-defined subgroup (where $$X=0$$) and $$j$$-th vaccinated subject ($$j=1,\ldots ,\,J$$) in the second covariate-defined subgroup (where $$X=1)$$, respectively. Let $${T}_{m}^{\text{control}}$$, $${T}_{n}^{\text{control}}$$ be the immunogenicity biomarker measurement for $$m$$-th control subject ($$m=1,\ldots ,\,M$$) in the first covariate-defined subgroup (where $$X=0$$) and $$n$$-th control subject ($$n=1,\ldots ,\,N$$) in the second covariate-defined subgroup (where $$X=1$$), respectively. The immunogenicity-based VE in subgroups of interest can be estimated using the subgroup-specific risk curves, $$\rho \left(T\right|X=0)$$, $$\rho \left(T\right|X=1)$$, obtained by covariate-adjusted models (Eqs. 1 and 2) and subgroup-specific immunogenicity data, $${T}_{i}^{\text{vaccinated}}$$, $${T}_{j}^{\text{vaccinated}}$$, $${T}_{m}^{\text{control}}$$, $${T}_{n}^{\text{control}}$$, as:

12 $${\rm{VE}}\left(T|X=0\right)=(1-{\rm{HR}})\cdot 100=\left(1-\frac{\frac{1}{I}{\sum }_{i=1}^{I}\rho ({T}_{i}^{\text{vaccinated}}{{|}}X=0)}{\frac{1}{M}{\sum }_{m=1}^{M}\rho ({T}_{m}^{\text{control}}{{|}}X=0)}\right)\,\cdot\, 100$$

13 $${\rm{VE}}(T|X=1)=(1-{\rm{HR}})\cdot 100=\left(1-\frac{\frac{1}{J}{\sum }_{j=1}^{J}\rho ({T}_{j}^{\text{vaccinated}}{{|}}X=1)}{\frac{1}{N}{\sum }_{n=1}^{N}\rho ({T}_{n}^{\text{control}}{{|}}X=1)}\right)\,\cdot\, 100$$

The VE point estimates in subgroups are obtained using the coefficient values, e.g., ${\beta }_{1},\,{\beta }_{2},\,{\beta }_{\mathrm{1,2}},\,\ldots ,\,{\beta }_{L}$, that were fit to the original observed time-to-event, immunogenicity, and demographic data. For a given set of data, the 95% CI associated with estimated VE needs to account for the uncertainty regarding the ${\beta }_{1},\,{\beta }_{2},\,{\beta }_{\mathrm{1,2}},\,\ldots ,\,{\beta }_{L}$ parameters and variability in the observed data. This can be done via parametric resampling of the posterior distribution for parameters and bootstrapping the observed data in the vaccinated and control groups. The bootstrap resampling of observed data is performed on subjects (1000 times): each time a subject is selected, his/her immunogenicity value and all his/her demographic characteristics (covariate values) are used (to account for the titer distribution and its uncertainty) in the estimation of VE. To account for uncertainty in the model parameters, for each of the 1000 bootstrapped data sets a set of model parameter values is randomly drawn from the parameters’ probability distribution (a multivariate normal distribution characterized by the coefficients’ point estimates and the covariance matrix derived from the fit). The 2.5th and 97.5th percentiles of these 1000 VE values are used to establish the 95% CI.

### Reporting summary

Further information on research design is available in the Nature Research Reporting Summary linked to this article.

## Supplementary information

Supplementary Information

## Data Availability

All data that support the findings in this work are available publicly from the cited references except for the subject-level data used in the zoster vaccine analysis. The data that support the findings in that section will be made available according to the data sharing policy of Merck Sharp & Dohme LLC, a subsidiary of Merck & Co., Inc., Rahway, NJ, USA, which is available at http://engagezone.msd.com/ds_documentation.php. Requests for access to the clinical study data can be submitted through the Engage Zone site or via email to Data Access mailbox.

## Code availability

The *vaxpmx* R package implementing proposed methods is available in the Comprehensive R Archive Network at https://cran.r-project.org/web/packages/vaxpmx/index.html. The R code supporting the conclusions of this article is available in the GitHub repository at https://github.com/MSDLLCpapers/usevaxpmx.
