# Supplementary material for: Improving precision of vaccine efficacy evaluation using immune correlate data in time-to-event models
Source: NPJ Vaccines. 2024 Nov 11;9:214. doi: 10.1038/s41541-024-00937-6 (PMC11554669; doi:10.1038/s41541-024-00937-6)
Supplement: Supplementary file 1 — Supplementary Information [file 41541_2024_937_MOESM1_ESM.pdf]

Supplementary Table 1: Assessment of correlate of risk and correlate of protection for zoster vaccine. P-values less than 0.1, 0.05, 0.01, 0.001 are denoted as "°", "*", "**" and "***", respectively. Even though the quadratic model (Model b) fit the data better than the linear model (AIC, quadratic, 426.0; AIC, linear, 428.0), the uncertainty in its coefficients was high (p-values, 0.226 and 0.143), likely due to relatively small dataset (32 HZ cases). Thus, the simpler, linear model (Model a) was selected for further analyses.

Model	Independent Variable	Coefficient	Standard Error (Coefficient)	P-Value	AIC
a:<br>HZ endpoint,<br>Cox PH model	Log_2 fold rise	-1.841	0.778	0.018 *	428.0
b:<br>HZ endpoint,<br>Cox PH model	Log_2 fold rise<br>$(Log_2 \text{ fold rise})^2$	3.876<br>-8.225	3.199<br>5.609	0.226<br>0.143	426.0
c:<br>HZ endpoint,<br>Cox PH model	Log_2 fold rise<br>Age group	-1.860<br>-0.320 (younger)	0.784<br>0.355	0.018 *<br>0.367	429.2
d:<br>HZ endpoint,<br>Cox PH model	Log_2 fold rise<br>Age group<br>Log_2 fold rise · age group	-2.561<br>-0.392 (younger)<br>1.094 (younger)	1.537<br>0.371<br>1.771	0.096 °<br>0.290<br>0.537	430.8
e:<br>HZ endpoint,<br>Cox PH model	Log_2 fold rise<br>Vaccination	-1.721<br>-0.129 (zoster vaccine)	0.867<br>0.448	0.047 *<br>0.772	429.9

Supplementary Table 2: Assessment of correlate of risk for CYD-TDV dengue vaccine. Best-fitting CoR models (Section 5.2) for each type of clinical endpoint (DENV-Any or serotype-specific DENV), modeling approach (Cox PH model, cause-specific Cox PH model, or Fine-Gray subdistribution hazards model), and immunogenicity predictor (principal component(s) or average titer). P-values less than 0.1, 0.05, 0.01, 0.001 are denoted as "°", "*", "**" and "***", respectively.

The proportional hazard assumption was satisfied for all listed models (p-values of the association between scaled Schoenfeld residuals [Schoenfeld, 1982; Grambsch & Therneau, 1994] with time were higher than 0.05). However, the relatively small p-values of the association between scaled Schoenfeld residuals with time (marginally significant, p-values were lower than 0.1, results not shown) for models 4a-I, 4c-I, and 4d-I signal potential non-proportionality of hazards, thus use of models with time-varying coefficients may be considered in future work.

Model	Independent Variable	Coefficient	Standard Error (Coefficient)	P-Value	AIC
0a-I: DENV-Any endpoint, Cox PH model, principal components	PC1<br>$PC1^2$<br>Serostatus	-0.523<br>-0.086<br>-0.987 (seropositive)	0.120<br>0.028<br>0.290	1.28e-05 ***<br>2.04e-03 **<br>6.71e-04 ***	940.4
0b-I: DENV-Any endpoint, Cox PH model, average titer	Average titer<br>Average $titer^2$<br>Serostatus	-1.042<br>-0.343<br>-0.988 (seropositive)	0.239<br>0.112<br>0.290	1.33e-05 ***<br>2.14e-03 **<br>6.70e-04 ***	940.5
1a-I: DENV1 endpoint, Cause-specific Cox PH model, principal components	PC1<br>Serostatus<br>PC1 · serostatus	0.159<br>-2.917 (seropositive)<br>-0.721 (seropositive)	0.246<br>0.817<br>0.310	0.516<br>3.54e-04 ***<br>0.020 *	125.8
1b-I: DENV1 endpoint, Cause-specific Cox PH model, average titer	Average titer<br>Serostatus<br>Average titer · serostatus	0.320<br>-2.919 (seropositive)<br>-1.445 (seropositive)	0.491<br>0.817<br>0.619	0.515<br>3.50e-04 ***<br>0.020 *	125.8
1c-I: DENV1 endpoint, Fine-Gray model, principal components	PC1<br>Serostatus<br>PC1 · serostatus	0.171<br>-2.918 (seropositive)<br>-0.729 (seropositive)	0.245<br>0.816<br>0.309	0.503<br>3.52e-04 ***<br>0.020 *	126.3
1d-I: DENV1 endpoint, Fine-Gray model, average titer	Average titer<br>Serostatus<br>Average titer · serostatus	0.342<br>-2.920 (seropositive)<br>-1.460 (seropositive)	0.490<br>0.816<br>0.619	0.501<br>3.48e-04 ***<br>0.020 *	126.3
2a-I: DENV2 endpoint, Cause-specific Cox PH model, principal components	PC1<br>$PC1^2$<br>Serostatus	-0.814<br>-0.173<br>-0.854 (seropositive)	0.196<br>0.051<br>0.398	3.40e-05 ***<br>7.32e-04 ***<br>0.032 *	467.0
2b-I: DENV2 endpoint, Cause-specific Cox PH model, average titer	Average titer<br>Average $titer^2$<br>Serostatus	-1.625<br>-0.691<br>-0.855 (seropositive)	0.392<br>0.205<br>0.398	3.44e-05 ***<br>7.46e-04 ***<br>0.032 *	467.0
2c-I: DENV2 endpoint, Fine-Gray model, principal components	PC1<br>$PC1^2$<br>Serostatus	-0.815<br>-0.174<br>-0.742 (seropositive)	0.197<br>0.051<br>0.393	6.08e-08 ***<br>2.04e-05 ***<br>0.057 °	471.1
2d-I: DENV2 endpoint, Fine-Gray model, average titer	Average titer<br>Average $titer^2$<br>Serostatus	-1.626<br>-0.696<br>-0.743 (seropositive)	0.394<br>0.205<br>0.393	6.23e-08 ***<br>2.28e-05 ***<br>0.057 °	471.1

3a-I:<br>DENV3 endpoint,<br>Cause-specific Cox PH model, principal components	PC1	-0.278	0.122	0.022 *	162.5
3b-I:<br>DENV3 endpoint,<br>Cause-specific Cox PH model,<br>average titer	Average titer	-0.556	0.244	0.023 *	162.5
3c-I:<br>DENV3 endpoint,<br>Fine-Gray model,<br>principal components	PC1	-0.235	0.124	0.044 *	166.8
3d-I:<br>DENV3 endpoint,<br>Fine-Gray model,<br>average titer	Average titer	-0.470	0.248	0.045 *	166.8
4a-I:<br>DENV4 endpoint,<br>Cause-specific Cox PH model, principal components	PC2<br>Serostatus	1.442<br>-2.276 (seropositive)	0.584<br>0.926	0.013 *<br>0.014 *	57.3
4b-I:<br>DENV4 endpoint,<br>Cause-specific Cox PH model,<br>average titer	Average titer	-0.783	0.382	0.040 *	61.1
4c-I:<br>DENV4 endpoint,<br>Fine-Gray model,<br>principal components	PC2<br>Serostatus	1.439<br>-2.100 (seropositive)	0.587<br>0.925	0.045 *<br>0.017 *	58.5
4d-I:<br>DENV4 endpoint,<br>Fine-Gray model,<br>average titer	Average titer	-0.747	0.385	0.061 *	61.9

Supplementary Table 3: Assessment of correlate of protection for CYD-TDV dengue vaccine (using Prentice criterion of independence of vaccination status when controlling for immunogenicity). Best-fitting CoR models (Table S2) with vaccination status as an additional predictor for each type of clinical endpoint (DENV-Any or serotype-specific DENV), modeling approach (Cox PH model, cause-specific Cox PH model, or Fine-Gray subdistribution hazards model), and immunogenicity predictor (principal component(s) or average titer). P-values less than 0.1, 0.05, 0.01, 0.001 are denoted as "°", "*", "**" and "***", respectively.

The proportional hazard assumption was satisfied for all listed models (p-values of the association between scaled Schoenfeld residuals [Schoenfeld, 1982; Grambsch & Therneau, 1994] with time were higher than 0.05). However, the relatively small p-values values of the association between scaled Schoenfeld residuals with time (lower than 0.1, results not shown) for models 0a-II, 0b-II, 4a-II, 4c-II, and 4d-II signal potential non-proportionality of hazards, thus use of models with time-varying coefficients may be considered in future work.

Model	Independent Variable	Coefficient	Standard Error (Coefficient)	P-Value	AIC
0a-II:<br>DENV-Any endpoint,<br>Cox PH model,<br>principal components	PC1<br>$PC1^2$<br>Serostatus<br>Vaccination	-0.516<br>-0.089<br>-1.031 (seropositive)<br>-0.103 (CYD-TDV)	0.121<br>0.029<br>0.314<br>0.279	1.95e-05 ***<br>1.98e-03 **<br>1.03e-03 **<br>0.711	942.2
0b-II:<br>DENV-Any endpoint,<br>Cox PH model,<br>average titer	Average titer<br>Average titer²<br>Serostatus<br>Vaccination	-1.030<br>-0.353<br>-1.030 (seropositive)<br>-0.101 (CYD-TDV)	0.242<br>0.115<br>0.314<br>0.280	2.03e-05 ***<br>2.10e-03 **<br>1.04e-03 **<br>0.719	942.3
1a-II:<br>DENV1 endpoint,<br>Cause-specific Cox PH<br>model, principal<br>components	PC1<br>Serostatus<br>PC1 · serostatus<br>Vaccination	0.163<br>-2.927 (seropositive)<br>-0.722 (seropositive)<br>-0.023 (CYD-TDV)	0.282<br>0.904<br>0.310<br>0.883	0.563<br>1.20e-03 **<br>0.020 *<br>0.980	127.8
1b-II:<br>DENV1 endpoint,<br>Cause-specific Cox PH<br>model,<br>average titer	Average titer<br>Serostatus<br>Average titer · serostatus<br>Vaccination	0.325<br>-2.927 (seropositive)<br>-1.446 (seropositive)<br>-0.018 (CYD-TDV)	0.564<br>0.904<br>0.621<br>0.884	0.564<br>1.20e-03 **<br>0.020 *<br>0.984	127.8
1c-II:<br>DENV1 endpoint,<br>Fine-Gray model,<br>principal components	PC1<br>Serostatus<br>PC1 · serostatus<br>Vaccination	0.174<br>-2.927 (seropositive)<br>-0.730 (seropositive)<br>-0.020 (CYD-TDV)	0.282<br>0.904<br>0.310<br>0.886	0.535<br>8.73e-04 ***<br>0.018 *<br>0.983	128.3
1d-II:<br>DENV1 endpoint,<br>Fine-Gray model,<br>average titer	Average titer<br>Serostatus<br>Average titer · serostatus<br>Vaccination	0.347<br>-2.927 (seropositive)<br>-1.461 (seropositive)<br>-0.016 (CYD-TDV)	0.564<br>0.904<br>0.620<br>0.887	0.535<br>8.70e-04 ***<br>0.018 *<br>0.987	128.3
2a-II:<br>DENV2 endpoint,<br>Cause-specific Cox PH<br>model, principal<br>components	PC1<br>$PC1^2$<br>Serostatus<br>Vaccination	-0.823<br>-0.171<br>-0.800 (seropositive)<br>0.127 (CYD-TDV)	0.198<br>0.052<br>0.431<br>0.397	3.40e-05 ***<br>1.07e-03 **<br>0.063 °<br>0.749	468.9
2b-II:<br>DENV2 endpoint,<br>Cause-specific Cox PH<br>model,<br>average titer	Average titer<br>Average titer²<br>Serostatus<br>Vaccination	-1.643<br>-0.681<br>-0.800 (seropositive)<br>0.130 (CYD-TDV)	0.397<br>0.209<br>0.431<br>0.398	3.43e-05 ***<br>1.10e-03 **<br>0.064 °<br>0.743	468.9
2c-II:<br>DENV2 endpoint,<br>Fine-Gray model,<br>principal components	PC1<br>$PC1^2$<br>Serostatus<br>Vaccination	-0.824<br>-0.172<br>-0.683 (seropositive)<br>0.142 (CYD-TDV)	0.199<br>0.052<br>0.425<br>0.399	1.53e-07 ***<br>4.17e-05 ***<br>0.106<br>0.733	472.9
2d-II:<br>DENV2 endpoint,<br>Fine-Gray model,<br>average titer	Average titer<br>Average titer²<br>Serostatus<br>Vaccination	-1.646<br>-0.685<br>-0.682 (seropositive)<br>0.145 (CYD-TDV)	0.398<br>0.209<br>0.425<br>0.399	1.54e-07 ***<br>4.75e-05 ***<br>0.107<br>0.727	473.0

3a-II:<br>DENV3 endpoint,<br>Cause-specific Cox PH model, principal components	PC1<br>Vaccination	-0.371<br>0.792 (CYD-TDV)	0.151<br>0.712	0.014 *<br>0.266	163.2
3b-II:<br>DENV3 endpoint,<br>Cause-specific Cox PH model,<br>average titer	Average titer<br>Vaccination	-0.742<br>0.793 (CYD-TDV)	0.302<br>0.712	0.014 *<br>0.266	163.2
3c-II:<br>DENV3 endpoint,<br>Fine-Gray model,<br>principal components	PC1<br>Vaccination	-0.307<br>0.661 (CYD-TDV)	0.147<br>0.682	0.032 *<br>0.326	167.8
3d-II:<br>DENV3 endpoint,<br>Fine-Gray model,<br>average titer	Average titer<br>Vaccination	-0.614<br>0.661 (CYD-TDV)	0.295<br>0.683	0.033 *<br>0.325	167.8
4a-II:<br>DENV4 endpoint,<br>Cause-specific Cox PH model, principal components	PC2<br>Serostatus<br>Vaccination	1.290<br>-2.613 (seropositive)<br>-2.320 (CYD-TDV)	0.532<br>1.002<br>1.131	0.015 *<br>9.15e-03 **<br>0.040 *	53.8
4b-II:<br>DENV4 endpoint,<br>Cause-specific Cox PH model,<br>average titer	Average titer<br>Vaccination	-0.440<br>-1.913 (CYD-TDV)	0.392<br>1.184	0.263<br>0.106	60.0
4c-II:<br>DENV4 endpoint,<br>Fine-Gray model,<br>principal components	PC2<br>Serostatus<br>Vaccination	1.297<br>-2.526 (seropositive)<br>-2.421 (CYD-TDV)	0.529<br>1.005<br>1.135	0.010 *<br>8.16e-03 **<br>0.020 *	54.5
4d-II:<br>DENV4 endpoint,<br>Fine-Gray model,<br>average titer	Average titer<br>Vaccination	-0.406<br>-1.932 (CYD-TDV)	0.392<br>1.180	0.343<br>0.135	60.7

Supplementary Table 4: Immunogenicity-based CYD-TDV dengue vaccine efficacy estimates in seropositive and seronegative subjects. For efficacy estimation the best-fitting models (I or II) for each type of clinical endpoint (DENV-Any or serotype-specific DENV), modeling approach (Cox PH model, cause-specific Cox PH, or Fine-Gray subdistribution hazards model), and immunogenicity predictor (principal component(s) or average titer) were used. DENV-4 models with principal components did not meet Prentice criterion, thus included vaccination status as a predictor.

Model	Independent Variable	AIC	Vaccine Efficacy (95% CI), Seropositive	Vaccine Efficacy (95% CI), Seronegative
0a-I: DENV-Any endpoint, Cox PH model, principal components	PC1<br>$PC1^2$<br>Serostatus	940.4	29 (13 to 44)	-14 (-155 to 43)
0b-I: DENV-Any endpoint, Cox PH model, average titer	Average titer<br>Average $titer^2$<br>Serostatus	940.5	29 (14 to 43)	-14 (-136 to 47)
1a-I: DENV1 endpoint, Cause-specific Cox PH model, principal components	PC1<br>Serostatus<br>PC1 · serostatus	125.8	63 (21 to 86)	-87 (-1568 to 67)
1b-I: DENV1 endpoint, Cause-specific Cox PH model, average titer	Average titer<br>Serostatus<br>Average titer · serostatus	125.8	63 (20 to 86)	-87 (-1404 to 70)
1c-I: DENV1 endpoint, Fine-Gray model, principal components	PC1<br>Serostatus<br>PC1 · serostatus	126.3	62 (23 to 86)	-95 (-1450 to 73)
1d-I: DENV1 endpoint, Fine-Gray model, average titer	Average titer<br>Serostatus<br>Average titer · serostatus	126.3	63 (19 to 85)	-96 (-1493 to 70)
2a-I: DENV2 endpoint, Cause-specific Cox PH model, principal components	PC1<br>$PC1^2$<br>Serostatus	467.0	32 (12 to 50)	-188 (-1077 to 25)
2b-I: DENV2 endpoint, Cause-specific Cox PH model, average titer	Average titer<br>Average $titer^2$<br>Serostatus	467.0	32 (12 to 51)	-187 (-1096 to 28)
2c-I: DENV2 endpoint, Fine-Gray model, principal components	PC1<br>$PC1^2$<br>Serostatus	471.1	31 (13 to 50)	-195 (-1101 to 14)
2d-I: DENV2 endpoint, Fine-Gray model, average titer	Average titer<br>Average $titer^2$<br>Serostatus	471.1	32 (12 to 49)	-194 (-1214 to 22)
3a-I: DENV3 endpoint, Cause-specific Cox PH model, principal components	PC1	162.5	31 (4 to 57)	64 (18 to 85)

3b-I:<br>DENV3 endpoint,<br>Cause-specific Cox PH model,<br>average titer	Average titer	162.5	32 (4 to 59)	64 (18 to 84)
3c-I:<br>DENV3 endpoint,<br>Fine-Gray model,<br>principal components	PC1	166.8	26 (0 to 55)	58 (3 to 83)
3d-I:<br>DENV3 endpoint,<br>Fine-Gray model,<br>average titer	Average titer	166.8	26 (2 to 53)	58 (0 to 83)
4a-II:<br>DENV4 endpoint,<br>Cause-specific Cox PH model, principal components	PC2<br>Serostatus<br>Vaccination	53.8	92 (29 to 99)	85 (-44 to 98)
4b-I:<br>DENV4 endpoint,<br>Cause-specific Cox PH model,<br>average titer	Average titer	61.1	45 (2 to 78)	76 (10 to 94)
4c-II:<br>DENV4 endpoint,<br>Fine-Gray model,<br>principal components	PC2<br>Serostatus<br>Vaccination	54.5	93 (47 to 99)	87 (-64 to 98)
4d-I:<br>DENV4 endpoint,<br>Fine-Gray model,<br>average titer	Average titer	61.9	43 (-1 to 80)	74 (-12 to 93)

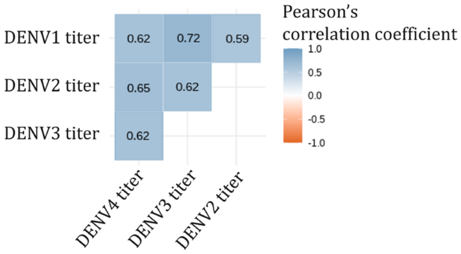

Supplementary Figure 1: Serotype-specific log_2 $PRNT_{50}$ titers are correlated. Pairwise Pearson's correlation coefficients of serotype-specific antibody titers (log_2 $PRNT_{50}$) at day 28 (i.e., 28 days post dose 3).

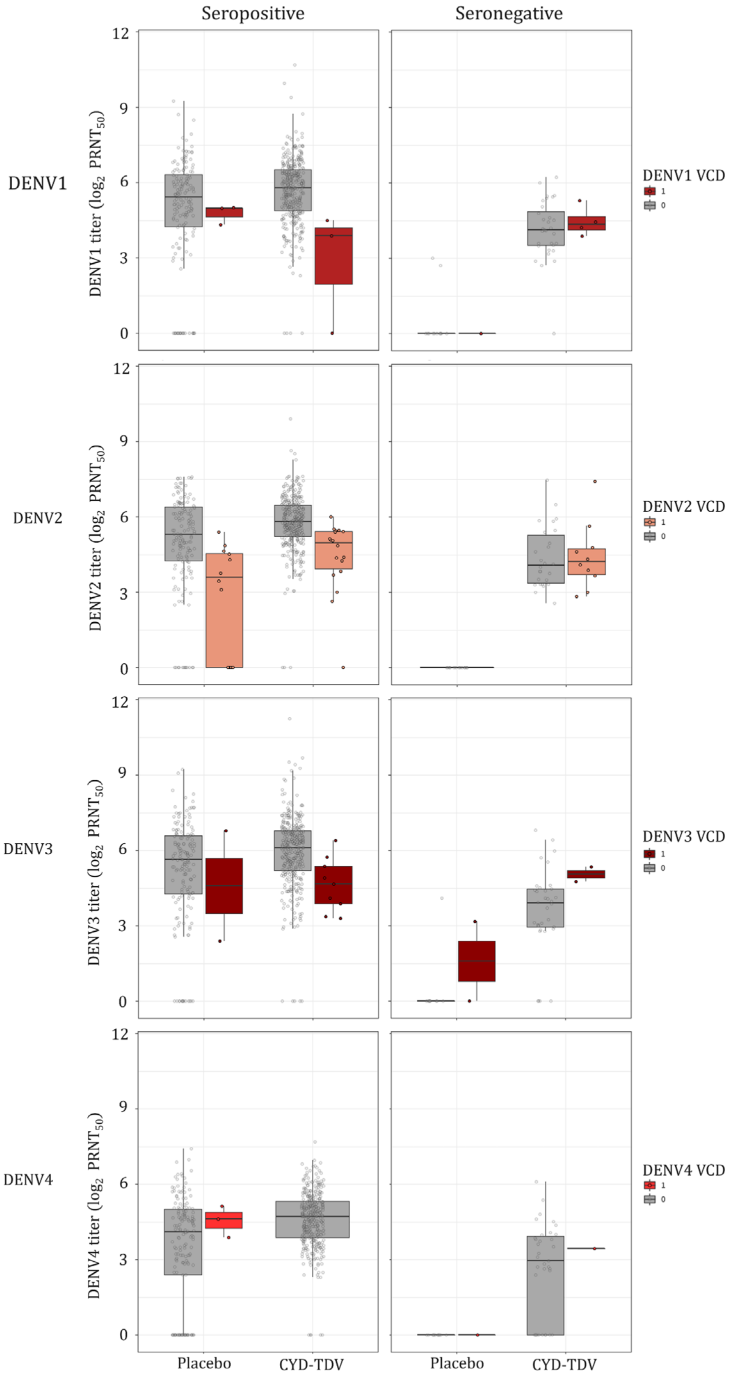

Supplementary Figure 2: CYD-TDV is generally less immunogenic in seronegative subjects than in seropositive subjects for all serotypes. Distribution of serotype-specific antibody titers (log$_2$ PRNT$_{50}$) at day 28 (i.e., 28 days post dose 3) in VCD cases (red circles) occurring between day 28 and end of follow-up (approximately 6 years), and non-cases (participants who did not have VCD during the follow-up) [Salje et al., 2021]. Boxplots show first quartile (lower edge of the box), median (horizontal line in the box), and third quartile (upper edge of the box), with participants stratified according to treatment assignment and serotype-specific VCD endpoint; the whiskers indicate 1.5 times the interquartile range from the box. Red and gray circles show individual serotype-specific day 28 antibody titers of serotype-specific VCD cases and non-cases, respectively.
